# Role of dietary and nutritional interventions in ceramide-associated diseases

**DOI:** 10.1016/j.jlr.2024.100726

**Published:** 2024-12-10

**Authors:** Shengnan Wang, Zihui Jin, Biyu Wu, Andrew J. Morris, Pan Deng

**Affiliations:** 1 Jiangsu Key Laboratory of Neuropsychiatric Diseases and College of Pharmaceutical Sciences, Soochow University, Suzhou, Jiangsu, China; 2 Department of Pharmacology and Toxicology, University of Arkansas for Medical Sciences and Central Arkansas Veterans Affairs Healthcare System, Little Rock, Arkansas, USA

**Keywords:** ceramide, dietary intervention, cardiometabolic disease, neurological disease, autoimmune, cancer

## Abstract

Ceramides are important intermediates in sphingolipid metabolism and serve as signaling molecules with independent biological significance. Elevated cellular and circulating ceramide levels are consistently associated with pathological conditions including cardiometabolic diseases, neurological diseases, autoimmune diseases, and cancers. Although pharmacological inhibition of ceramide formation often protects against these diseases in animal models, pharmacological modulation of ceramides in humans remains impractical. Dietary interventions including the Mediterranean diet, lacto-ovo-vegetarian diet, calorie-restricted diet, restriction of dairy product consumption, and dietary supplementation with polyunsaturated fatty acids, dietary fibers, and polyphenols, all have beneficial effects on modulating ceramide levels. Mechanistic insights into these interventions are discussed. This article reviews the relationships between ceramides and disease pathogenesis, with a focus on dietary intervention as a viable strategy for lowering the concentration of circulating ceramides.

Over the past few decades, ceramides have received particular attention for their critical role in a myriad of cellular and physiological processes, including cell growth, differentiation (1), senescence, apoptosis (2), vesicle trafficking (3, 4), and the stress response (5). Ceramides are also known for their physiological function of maintaining skin moisture by forming a reticular structure in the stratum corneum (6, 7). In addition, ceramides are key lipotoxic players in metabolism, and compelling evidence has linked elevated ceramides to insulin resistance (IR), dyslipidemia, and cell death, which can lead to cancer (8), inflammation (9), cardiometabolic diseases (10, 11), autoimmune diseases (12), and neurological diseases (13). Studies assessing ceramide levels in tissues (liver, fat, and skeletal muscle) have revealed a positive correlation between ceramides and IR (14, 15, 16), and circulating ceramides have been identified as disease biomarkers (17). For example, the Mayo Clinic has developed a clinical test involving measurements of plasma ceramide C16:0, C18:0, and C24:1 levels, as well as the ratio of these three ceramides to C24:0 ceramides, to predict major adverse cardiovascular events (18). Furthermore, an elevated C18:0/C16:0 ceramide ratio has been proven to be an independent marker of diabetes risk (19).

The role of ceramide in diseases has been extensively reviewed elsewhere (17, 20, 21, 22). Preclinical studies have demonstrated the protective effects of inhibiting ceramide synthesis against cardiometabolic diseases (23, 24, 25), neurodegenerative diseases (26), and cancer (27, 28). Consequently, ceramides and associated pathways are emerging as promising therapeutic targets for these diseases (29, 30, 31, 32, 33, 34). However, the lack of pharmacological agents capable of safely and effectively modulating ceramide levels in humans poses a significant challenge. Recent studies have suggested that dietary interventions hold promise for altering circulating ceramide concentrations. Examples include the Mediterranean diet (MD) (35), lacto-ovo-vegetarian diet (36), calorie-restricted diet (37), dietary fibers (38), PUFAs (39), and polyphenols (40). These dietary interventions can prevent or ameliorate to some extent abnormal physiological activities and diseases associated with elevated levels of circulating ceramides (12, 41). This review provides a comprehensive overview of ceramide-associated diseases, including cardiometabolic diseases, neurological diseases, autoimmune diseases, and cancer. Notably, nutritional intervention approaches are highlighted, providing a scientific basis for the design of precision-based nutritional interventions aimed at reducing ceramide levels in affected populations.

## Ceramide biosynthesis pathways

**Fig. 1:**
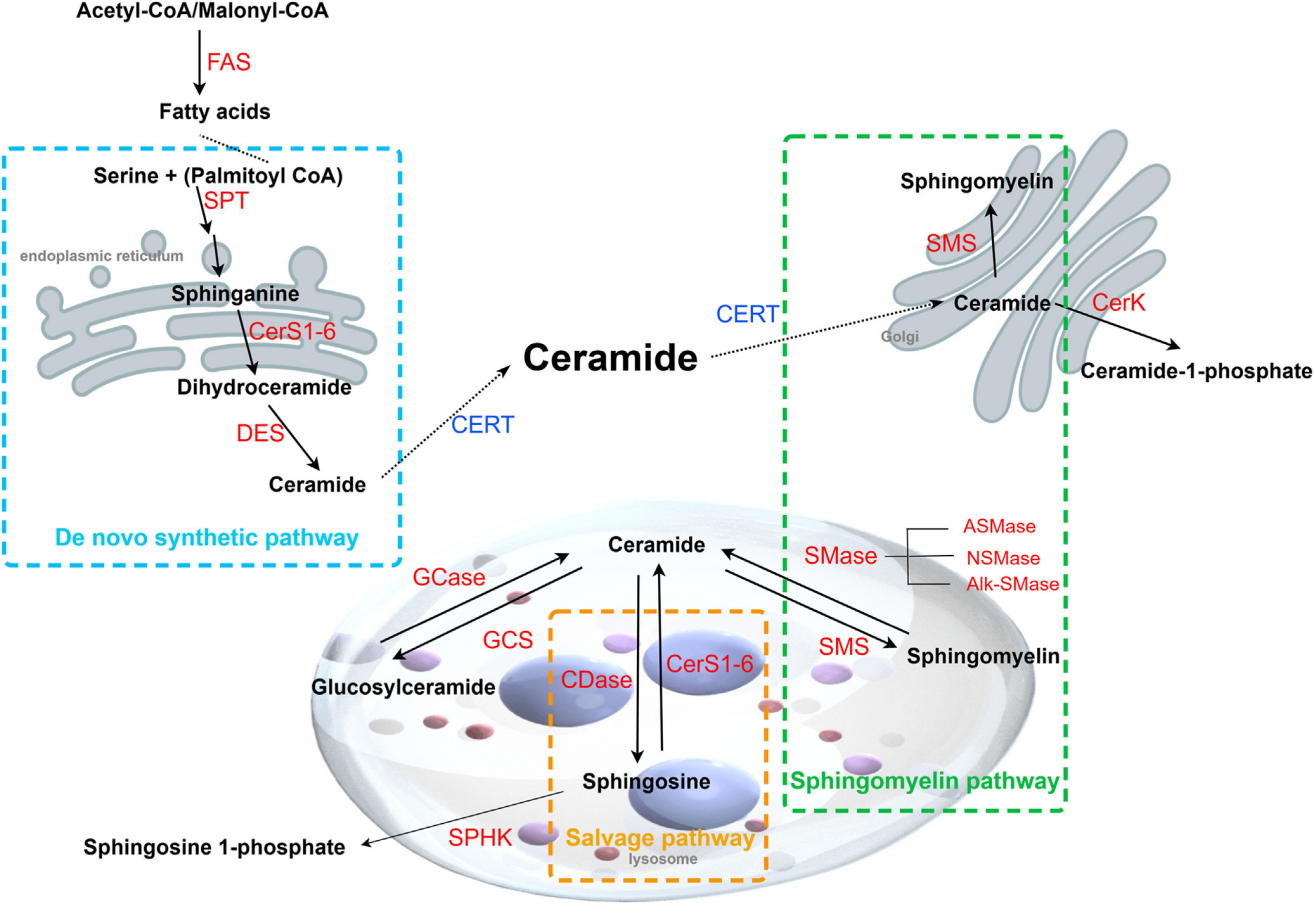
Ceramide biosynthesis pathways (created by Figdraw 2.0). Ceramides are central to sphingolipid metabolism and can be synthesized through three pathways. The condensation of serine and palmitoyl-CoA initiates the de novo synthesis pathway (blue box). Sphingomyelin can be hydrolyzed to produce ceramide (green box). Ceramide can be hydrolyzed to sphingosine and then reacylated back to ceramide in the salvage pathway (orange box). Alk-SMase, alkaline sphingomyelinase; CDase, ceramidase; CerK, ceramide kinase; CERT, ceramide transfer protein; DES, dihydroceramide desaturase; GBA, glucocerebrosidase; GCase, glucocerebrosidase; GCS, glucosylceramide synthase; SMase, sphingomyelinase; SMS, sphingomyelin synthase; SPHK, sphingosine kinase.

Ceramides are a complex class of sphingolipids located at the center of sphingolipid biosynthesis and metabolism. They are composed of a sphingosine backbone attached to FA chains of different lengths (C14–C36) by amide bonds. Three pathways contribute to ceramide biosynthesis (Fig. 1): *1*) de novo synthesis pathways, which are initiated by palmitoyl-CoA and serine within the endoplasmic reticulum; *2*) the hydrolysis pathway of SM, which occurs within multiple cellular compartments, including the plasma membrane, lysosomes, Golgi apparatus, and mitochondria; and *3*) the salvage pathway, which converts complex sphingolipids into sphingosine and generates ceramides through reacylation reactions within lysosomes and endosomes (42). Ceramides are produced from sphingosine and acyl-CoA by the action of ceramide synthase (CerS). CerSs are a family of six enzymes (CerS1-6) that are located mainly in the endoplasmic reticulum and have different strengths in the production of ceramides with varying chain lengths. CerS differs in selectivity for acyl-CoA depending on the length of the acyl chain (43). Although classifications may differ among researchers, ceramides are generally categorized by fatty acid acyl chain length into long-chain (C14-C20), very-long-chain (>C20:0), and ultra-long-chain (>C24:0) types (44). CerS1 preferentially synthesizes C18 ceramide; CerS4 synthesizes C18/C20/C24 ceramides; CerS5 and CerS6 preferentially synthesize C14/C16/C18 ceramides; CerS2 preferentially synthesizes C22/C24 ceramides; and CerS3 synthesizes ultra-long-chain ceramides (>C26) (21, 45). Ceramide transfer protein (CERT) is a ceramide carrier that is essential for ceramide and SM balance in cells (46). CERT is the only lipid transporter known to specifically deliver ceramides from the endoplasmic reticulum to the Golgi apparatus (47). In addition to sphingomyelin synthase and sphingomyelinase, CERT is another key factor that regulate the ceramide/sphingomyelin ratio (48).

## Ceramides and diseases

**Fig. 2:**
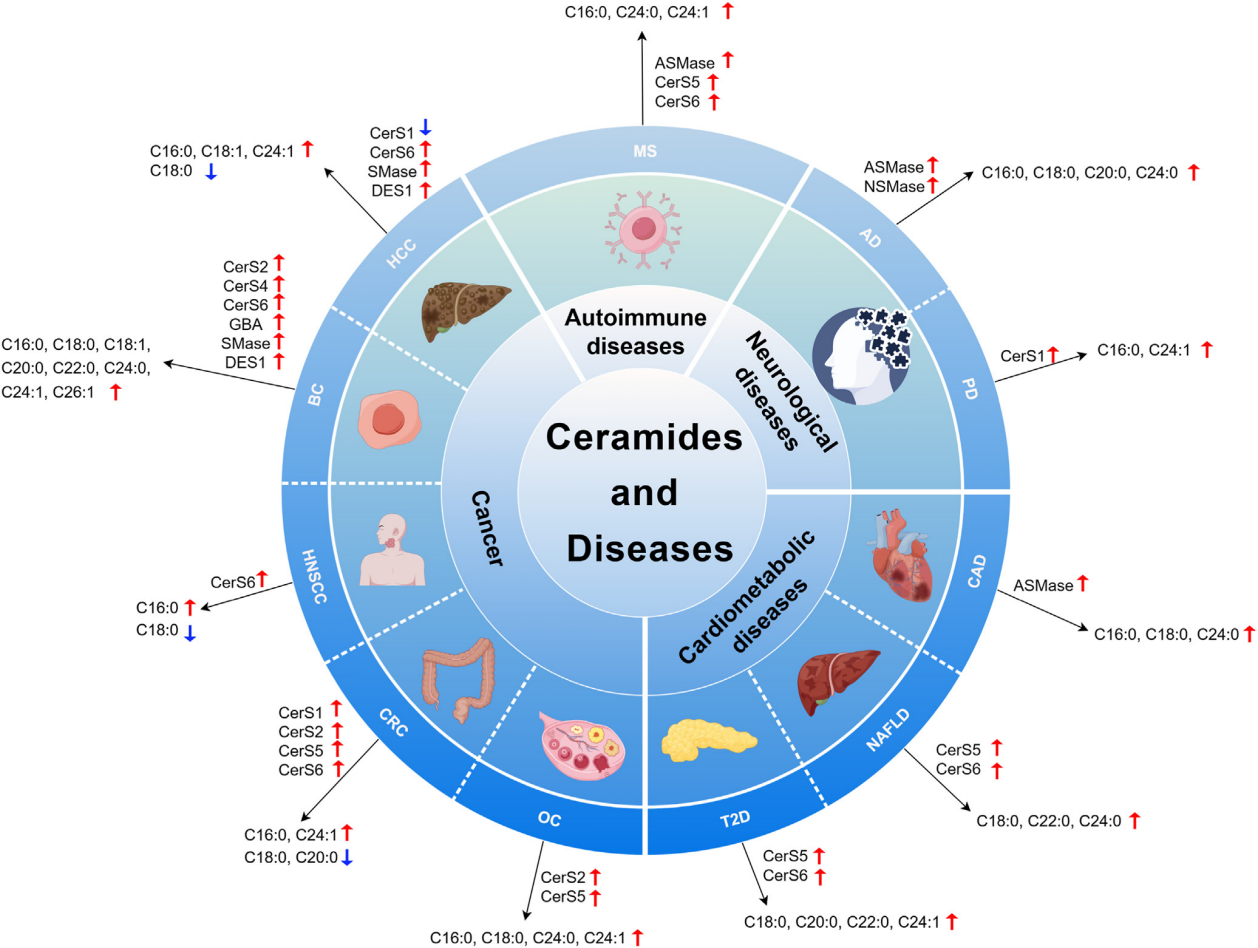
Alteration of ceramide levels and associated enzymes in diseases (created by Figdraw 2.0). Red arrows represent the upregulation of ceramide levels and enzymes, and blue arrows represent the downregulation of ceramide levels and enzymes. CAD, coronary artery disease; CRC, colorectal cancer; DES1, dihydroceramide desaturase 1; GBA, glucocerebrosidase; HCC, hepatocellular carcinoma; NAFLD, nonalcoholic fatty liver disease; OC, ovarian cancer; PD, Parkinson's disease; SMase, sphingomyelinase.

Ceramide elevation is a hallmark of numerous pathological conditions, including cardiometabolic diseases, neurological disorders, autoimmune diseases, and cancer. In this context, we summarize alterations in ceramide metabolism and ceramide levels in these diseases (Fig. 2), thus emphasizing the potential of ceramide pathways as targets in the development of intervention strategies aimed at ameliorating diseases associated with increased ceramide levels. Although clinical studies have shown promising results in suppressing ceramide and related metabolite formation using available medicines, such as liraglutide (25), fenofibrate (49), and rosuvastatin (50), several challenges remain in the development of new ceramide-lowering therapeutics. Key obstacles include off-target effects and compensatory activation of alternative pathways when specific enzymes are inhibited. For example, ABC294640 is a first-in-class orally available inhibitor of sphingosine kinase-2. However, blood and lymphatic system disorders were observed in the clinical trials of ABC294640 in refractory/relapsed multiple myeloma (NCT02757326). Prell *et al.* (51) recently reported significant off-target effects of ABC294640 on sphingolipid metabolism in multiple cell models. Furthermore, all seven tested sphingosine-1-phosphate (S1P) inhibitors exhibited unexpected on-target and/or off-target effects. In another study, a potent *N*-acetylgalactosamine-conjugated antisense oligonucleotide was developed to inhibit ceramide synthase 2 within hepatocytes (29). While it effectively reduces specific ceramides associated with an elevated risk of cardiovascular mortality, it simultaneously increases the levels of other ceramides linked to cardiovascular death (29). Therefore, ongoing research is essential for overcoming these challenges and advancing ceramide-based therapies toward safe and effective treatments.

### Cardiometabolic diseases

Cardiometabolic diseases, including IR, diabetes, heart attack, and nonalcoholic fatty liver disease (NAFLD), are the leading causes of morbidity and mortality globally (52). Ceramides are important molecular intermediates that link pathological metabolic stresses (i.e., glucocorticoids and saturated fats) to the initiation of IR (53). In humans, elevated circulating ceramide levels are associated with a higher risk of cardiometabolic diseases (54, 55, 56, 57, 58, 59, 60, 61, 62, 63), with the ratios between long- and very-long-chain ceramides in plasma, notably C16:0/C24:0, being more closely related to disease development (Table 1). Compared with healthy controls, obese individuals with IR exhibited nearly 2-fold higher total skeletal muscle ceramide content (46 ± 9 vs. 25 ± 2 pmol/2 mg muscle, *P* < 0.05) (16). In addition, a significant negative correlation (r = −0.49, *P* = 0.01) between total skeletal muscle ceramide content and insulin sensitivity in humans has been reported (64). Acid sphingomyelinase (ASMase) can convert SM to ceramides (70), and the expression and activity of this enzyme were found to be elevated in the adipose tissue of adult and pediatric obese patients (71, 72), as well as in the serum of patients with T2D (72), suggesting that elevated ASMase activity may contribute to increased ceramide levels in individuals with metabolic disorders. *CERS6* mRNA expression and C16:0 ceramide levels are elevated in adipose tissue in obese humans and are associated with IR (73). CerS5 and CerS6 preferentially incorporate palmitate into dihydroceramides, and the upregulation of these isoforms in T2D promotes dihydroceramide synthesis and leads to IR in the event of an oversupply of dietary palmitate (65). Furthermore, serum C16:0 and C18:0 ceramides are associated with markers of NF-*κ*B activation in muscle, suggesting that specific ceramide species can trigger intracellular inflammation (65). Notably, C16:0 and C18:0 ceramides are closely related to obesity because they regulate fatty acid oxidation and insulin signaling pathways (73, 74).

**Table 1:** Summary of studies assessing ceramide levels in patients with cardiometabolic diseases

Disease	Participants	Ceramide	Type of sample	Refs
IR	Individuals with BMI <27 kg/m^2^ were classified as lean control subjects (n = 10). Subjects with a BMI of 27–33 kg/m^2^ were classified as obese without diabetes (n = 10)	The total muscle ceramide content was nearly 2-fold (46 ± 9 vs. 25 ± 2 pmol/2 mg muscle, *P* < 0.05)	Human muscle tissue	(16)
IR risk	Male subjects between 20 and 40 years of age (n = 27)	There was a significant negative correlation between muscle total ceramide content and insulin sensitivity (r = −0.49, *P* = 0.01)	Human muscle tissue	(64)
T2D	FINRISK 2002 (n = 8,045), Western Norway Coronary Angiography Cohort (n = 3,344)	Ceramide ratios: C18:0/C16:0 ceramide increased	Human plasma	(19)
T2D	Obese sedentary controls (n = 14), T2D patients (n = 15), and endurance training athletes (n = 15)	C18:0, C20:0, and C24:1 ceramides increased	Human serum	(65)
T2D	T2D patients (n = 13), healthy nondiabetic control subjects (n = 14)	C18:0, C20:0, C24:1 ceramides increased	Human serum	(54)
T2D	Cardiovascular health study participants (n = 3,645)	C16:0, C18:0, C20:0, C24:1 ceramides increased	Human serum	(55)
T2D	Strong Heart Study (n = 435) and Strong Heart Family Study (n = 1902) participants	C18:0, C20:0, C22:0 ceramides increased	Human serum	(56)
T2D, CVD	Strong Heart Study (n = 267) and Strong Heart Family Study (n = 597) participants	Higher plasma C16:0 ceramide levels in patients with T2D are associated with a higher risk of CVD	Human blood	(57)
T2D	T2D patients (n = 335)	There was an independent association between liver fat content and plasma levels of C18:0, C20:0, C22:0, and C24:0 ceramides	Human plasma	(58)
CAD	Familial CAD (n = 462) and population-based controls (n = 212)	C18:0, C22:0, C24:0 ceramides increased	Human serum	(59)
CAD	CAD (n = 265) and no CAD (n = 230)	C16:0, C18:0, C24:1 ceramides increased	Human plasma	(18)
CAD	Subjects in the Corogene (n = 160), Special Program University Medicine—Inflammation in Acute Coronary Syndromes (n = 1,637), and Bergen Coronary Angiography Cohort studies (n = 81)	Ceramide ratios: C16:0/C24:0, C18:0/C24:0, C24:1/C24:0 ceramide increased	Human plasma	(60)
CHD, HF	Framingham Heart Study (n = 2,642) and study of Health in Pomerania (n = 3,134) participants	C24:0/C16:0 ceramide ratios were inversely correlated with coronary heart disease events, and C24:0/C16:0 and C22:0/C16:0 ceramide ratios were negatively correlated with all-cause mortality.	Human serum	(61)
Heart failure (HF)	Cardiovascular Health Study participants (n = 4,249)	Higher levels of C16:0 ceramide in plasma are associated with an increased risk of HF, whereas higher levels of C22:0 ceramide are associated with a reduced risk of HF	Human serum	(62)
Hypertensive patients at high CV risk	Hypertensive patients aged 18–85 years with high or very high cardiovascular risk (n = 225)	C16:0, C22:0, and C24:0 ceramides have a significant significance in predicting major adverse cardiovascular event	Human blood	(63)
NAFLD	Obese children (n = 80), including children with NAFLD (n = 31)	C14:0, C16:0, C16:1, C18:0, C18:1, C22:0, C24:0 ceramides increased	Human serum	(66)
NAFLD	Individuals with liver histological classification of normal (n = 31), steatosis (n = 17), NASH (n = 20), or cirrhosis (n = 20)	Plasma: C18:0, C18:1, C20:0, C22:0, C24:0, C24:1 ceramides increased; liver:C18:0, C20:0, C22:0, C24:0 ceramides increased	Human liver and plasma	(67)
NASH	Morbidly obese women (n = 46), of which NASH patients (n = 22)	Ceramides increased	Human serum and portal vein blood	(68)
IR, NAFLD	HOMA-IR >3.19 (n = 62), HOMA-IR ≤3.19 (n = 63)	C16:0, C18:0, C19:0, C20:0, C24:1 ceramides increased	Human liver	(14)
NASH	Obese (BMI >30 kg/m^2^) patients (n = 21) and 7 control subjects. Liver histology classifies obese patients into NAFL^−^ (n = 7), NAFL^+^ (n = 7), and NASH (n = 7)	Liver: ceramides, dihydroceramide, and lactosylceramides increased; serum: dihydroceramide increased	Human liver and serum	(69)

HF, heart failure; HOMA-IR, homeostasis model assessment of IR; NAFL^−^, obese patients without steatosis; NAFL^+^, obese patients with steatosis; NAFLD, nonalcoholic fatty liver disease; NASH, nonalcoholic steatohepatitis.

Ceramides also contribute significantly to the development and progression of NAFLD (75). Studies have revealed markedly elevated ceramide levels in the livers of patients with NAFLD (66, 67) and nonalcoholic steatohepatitis (68, 69) (Table 1). Furthermore, these elevated ceramide levels are positively correlated with the extent of oxidative stress and inflammation in the liver (76). Notably, the function of ceramides in NAFLD is also closely related to the length of their acyl chains. C16:0 ceramide, synthesized by CerS5 and CerS6, promotes endoplasmic reticulum stress, resulting in impaired liver function and perturbed metabolic homeostasis in patients (77). In contrast, CerS2-mediated synthesis of ultra-long-chain ceramides prevents palmitic acid-induced hepatocyte lipotoxicity and endoplasmic reticulum stress, consequently attenuating IR levels and hepatic steatosis (78, 79, 80). Serum ASMase levels are upregulated in patients with chronic liver disease, leading to elevated ceramide levels (81). In addition, both neutral sphingomyelinase (NSMase) and ASMase can be activated by proinflammatory conditions, further contributing to the progression of NAFLD.

### Neurological diseases

Abnormal ceramide metabolism has emerged as a critical factor in the pathogenesis of neurological diseases (82), including Alzheimer's disease (AD) (83, 84, 85, 86, 87, 88), Parkinson's disease (PD) (89, 90, 91), and mental disorders (92) (Table 2). Elevated levels of C16:0, C18:0, C20:0, and C24:0 ceramides were found in the brains of patients with AD and neurological deficits (94). Higher C22:0 and C24:0 ceramide levels predict cognitive decline and hippocampal volume loss in patients with amnesic mild cognitive impairment (106). In addition, high C16:0 and C24:0 ceramide levels have been identified as predictors of AD development (96). Notably, the highest ceramide levels were observed in patients displaying multiple neuropathological abnormalities (94). Ceramides are precursors to SM synthesis and can be formed by the hydrolysis of SM. Analysis of SM/ceramide and dihydrosphingomyelin/dihydroceramide ratios revealed that higher ratios were predictive of slower cognitive decline (95). In AD, the amyloid precursor protein undergoes a series of enzymatic steps to be processed into the amyloid-*β* (Aβ) peptide, which is secreted outside the cell (107). Neuronal exposure to Aβ directly triggers the increases in ceramide levels by activating NSMase (108). Filippov *et al.* (94) showed that ASMase and NSMase2 are upregulated in the brain tissue of patients with AD and neurological deficits (e.g., frontotemporal lobe degeneration, tauopathy, or diffuse Lewy body disease), which may account for or contribute to increased brain ceramide levels. Notably, ceramide accumulation in the brain can occur during the normal aging process. For example, C24:0 ceramide levels naturally increase in normal aging brain tissue (93, 109). In addition, gender-dependent relationships between ceramides and mild cognitive impairment/AD have been proposed, with associations more evident in males than females (97, 98). These studies highlight the importance of considering age- and sex-related factors in investigations concerning the relationship between ceramides and neurological diseases.

**Table 2:** Summary of studies assessing ceramide levels in patients with neurological diseases

Disease	Participants	Ceramide	Type of sample	References
AD	Human: AD group (n = 7), control group (n = 7); mice: 3, 6, 25 months (n = 5) male C57BL/6 mice	C24:0 ceramides increased	Human and mouse brain tissue	(93)
AD	CDR 0 (n = 5), CDR 0.5 (n = 3), CDR (n = 4), CDR (n = 6), and CDR (n = 4) white matter and gray matter samples in the brain and cerebellum of participants	AD white matter of the brain and cerebellum: C18:0 and C24:1 ceramides increased	Human brain tissue	(84)
AD	Patients with AD (n = 19), AD with other neuropathological lesions (n = 6), and non-AD dementia (n = 9). Control group (n = 6)	C16:0, C18:0, C20:0, and C24:0 ceramides increased	Human brain tissue	(94)
AD	Patients with AD (n = 120)	The ratio of SM/ceramide can predict clinical progression	Human plasma	(95)
AD	Participants without dementia (n = 72), with any dementia type (n = 27), and with AD (n = 18)	C16:0 and C24:0 ceramides increased	Human serum	(96)
AD	AD (n = 91), MCI (n = 92), and control (n = 26) participants	Ceramide (d38:4) increased	Human cerebrospinal fluid	(85)
MCI	MCI (n = 197) and control (n = 200) participants	The association of plasma C18:0 and C24:1 ceramide levels with MCI was regulated by sex but not by age, with higher MCI levels in males. No association was found in women	Human plasma	(97)
AD	AD (n = 20) and control (n = 20) participants	C24:1 ceramide increased	Human plasma	(86)
AD	Participants included men (n = 626) and women (n = 366)	In men, C16:0, C18:0, C22:0, C24:0, and C24:1 ceramides are associated with an increased risk of AD. In women, there was no association between ceramide and AD risk	Human plasma	(98)
AD	Preclinical AD (n = 12), patients with MCI due to AD (MCI-AD, n = 31), and healthy controls (n = 20)	Preclinical AD vs. healthy controls: ceramides increased	Human plasma	(87)
PD	Cognitively normal PD patients (n = 26), PD patients with cognitive impairment or dementia (n = 26), and cognitively normal non-PD controls (n = 5)	Among PD patients, levels of C16:0, C18:0, C20:0, C22:0, and C24:1 ceramide increased	Human plasma	(99)
PDD	PDD (n = 38), PD-NC (n = 40), and normal controls (n = 40)	C14:0 and C24:1 levels were significantly higher in PDD than in PD-NC and normal controls	Human plasma	(100)
PD	PD (n = 9) and controls (n = 10). Take the ACC and occipital cortex of the brain	PD ACC: overall gray matter ceramide decline; C16:0, C18:0, and C18:0 ceramides decline; C23:0 and C24:1 ceramides increased The gene expression of *CERS1* was upregulated	Human brain tissue	(101)
PD	PD (n = 36) and healthy controls (n = 36)	C16:0 ceramide increased	Human plasma	(90)
PD	PD (n = 50) and healthy controls (n = 45)	Ceramide (d40:0), Ceramide (d42:0) decreased	Human serum	(102)
PD	Control group (n = 63 serum and n = 20 control group) (n = 63 serum and n = 20 CSF), LRRK2 G2019S carriers without PD (n = 56 serum and n = 20 CSF), LRRK2 G2019S carriers diagnosed with PD (n = 65 serum and n = 19 CSF), and PD patients without known LRRK2 mutations (n = 37 serum and n = 29 CSF)	Serum of PD patients: ceramide (d18:1/24:0), ceramide (d18:2/24:0) decline, C16:0 ceramide increased LRRK2 G2019S carriers of CSF: ceramide (d32:1) decreased	Human serum and cerebrospinal fluid	(91)
Depression	The Erasmus Rucphen Family study (n = 901)	C20:0 and C22:0 ceramides were associated with the onset of depression	Human plasma	(103)
Depression	Recent-Depression (n = 5 NC and n = 10 AD), Past-Depression (4 NC and 4 AD), and No-Depression (16 NC and 7 AD)	Recent-Depression:C16:0, C18:0, C20:0, C24:1, and C26:1 ceramides increased	Human plasma	(104)
Depression, BD	Depression (n = 174), BD (n = 67)	C24:1 ceramide was the most important differential biomarker between BD and depression	Human dried blood spot	(105)

BD, bipolar disorder; CDR, clinical dementia rating; CSF, cerebrospinal fluid; LRRK2, leucine-rich repeat kinase 2; MCI, mild cognitive impairment; NC, no cognitive impairment; PD-NC, PD with no cognitive impairment; PDD, Parkinson's disease dementia.

PD is the second most common neurodegenerative disease after AD (110). Ceramides play a crucial role in PD pathogenesis, influencing the development and progression of the disease by modulating mitophagy processes (111). Compared with those with normal cognitive function, subjects with PD exhibited elevated plasma levels of ceramides C16:0, C20:0, C22:0, C24:1, and C26:1 (99). Xing *et al.* (100) reported significantly higher plasma levels of ceramide C14:0 and C24:1 in Parkinson's disease dementia patients than in both PD patients without cognitive impairment and healthy controls. Notably, the increased ceramide levels in Parkinson's disease dementia correlated with diminished memory function (100). A case-control analysis of sphingolipids in the anterior cingulate gyrus (ACC) and occipital cortex of postmortem brain tissue from patients with PD revealed that the C18:0 ceramide content in the ACC of PD patients was significantly higher than that in the control group without any significant neuropathology, whereas the C24:1 ceramide level was significantly lower in the PD group (101). Interestingly, these regional differences were not observed in the occipital cortex samples. This study also revealed alterations in the ceramide acyl chain composition within the ACC, where more long-chain ceramides (e.g., C16:0, C18:0, and C18:1) at the expense of very-long-chain ceramides (e.g., C23:0 and C24:1) were observed. This finding suggested a potential shift toward shorter ceramide acyl chain lengths in PD patients (101). Moreover, compelling evidence suggests that the interaction between glucocerebrosidase activity, *α*-synuclein, and PINK1 regulates ceramide metabolism in PD (102, 111, 112, 113). However, the precise mechanisms and functional implications of altered ceramide levels in PD warrant further investigations.

Abnormal ceramide levels have been implicated in mood disorders. Plasma levels of C20:0 and C22:0 ceramides were positively correlated with depression scores (103). In addition, elevated levels of C16:0, C18, C20, C24:1, and C26:1 ceramides were detected in the plasma of patients with depression (104). Notably, the C24:1 ceramide level showed promise in distinguishing depression from bipolar disorder, a condition often misdiagnosed as depression. (105). Detailed information on ceramides in neurological disease studies is summarized in Table 2.

Extracellular vesicles (EVs) have emerged as critical mediators in the pathogenesis of neurodegenerative diseases due to their role in transporting bioactive molecules, including neurotoxic factors such as Aβ and ceramides (114, 115). EVs isolated from cerebrospinal fluid of AD patients have been shown to contain high levels of Aβ (116). The lipid composition of EVs, particularly ceramides, influences their capacity to carry Aβ, as ceramides increase the biogenesis and secretion of Aβ-containing vesicles (117). Notably, Bieberich’s group reported that amyloid-induced astrocyte-derived EVs were rich in ceramide species (115). Furthermore, inhibition of NSMase, a key enzyme in ceramide production, suppresses the spread of neuronal exosomes and significantly reduces Aβ pathology in female AD mouse models, offering protection against cognitive dysfunction (118). These findings highlight EV-associated ceramide pathways as promising therapeutic targets for the treatment of neurodegenerative diseases (119).

### Autoimmune diseases

Autoimmune rheumatic diseases are a group of connective tissue disorders that primarily affect the musculoskeletal system. The role of ceramides in autoimmune rheumatic diseases has been extensively reviewed by Alexandropoulou *et al.* (12). Multiple sclerosis (MS) is a chronic inflammatory autoimmune disease characterized by immune cells infiltration into the central nervous system. Studies have consistently reported elevated plasma ceramide levels in MS patients (120, 121, 122) (Table 3). Specifically, the C16:0 ceramide level was significantly correlated with the Expanded Disability Status Scale (124, 126), a commonly used scale for assessing the level of disability in people with MS (129). Elevated levels of C16:0 and C18:0 ceramides are evident in demyelinated plaques within the central nervous system of MS patients compared with those in matched controls or autopsy normal-looking white matter samples from patients with neurological disorders of PD and encephalitis (125). In addition, elevated levels of C16:0 and C24:0 ceramides have been detected in the cerebrospinal fluid of patients with MS (120). Interestingly, Martynova *et al.* (128) reported seasonal variations in serum ceramide levels in MS patients. Unsupervised machine learning analysis of serum lipids identified ceramides as the first-line candidates for MS biomarkers (123), and the increase in ceramides was related to enhanced plasma ASMase activity in MS patients (127).

**Table 3:** Summary of studies assessing ceramide levels in patients with MS

Disease	Participants	Ceramide	Type of sample	References
MS	MS (n = 8), controls (n = 8)	C16:0 and C24:0 ceramides increased	Human cerebrospinal fluid	(120)
MS	MS (n = 102), controls (n = 301)	Unsupervised machine-learning analysis identified ceramides as first-line candidates for MS biomarkers	Human serum	(123)
MS	Women with MS were monitored in the first trimester (T1), second trimester (T2), third trimester (T3), and postpartum (n = 12)	T1 and T2:C16:0 ceramides increased. In the postpartum period, C16:0 and C18:0 ceramides were significantly associated with the EDSS	Human serum	(124)
MS	MS (n = 72), controls (n = 25)	C16:0 and C24:1 ceramides increased; *CERS2* and *CERS6* mRNA expression was upregulated	Human serum	(121)
MS	MS (n = 13), OND (n = 15)	Active lesions: ceramides increased; inactive lesions: ceramides decreased	Human autopsy brain	(125)
MS	MS (n = 251), controls (n = 68)	C16:0 and C22:1 ceramides increased baseline levels of Cer16:0 ceramide correlated with the chance of worsening EDSS at five years	Human serum	(126)
MS	MS (n = 18), controls (n = 18)	Ceramide and activity of ASMase increased	Human plasma	(127)
MS	Patients with MS (n = 33), patients with an unclassified CNS demyelinating disorder not matching the diagnostic criteria of MS (n = 6) and a control group (n = 40)	C16:0 ceramide increased; long-chain (18, 19, 20) and very long-chain (22, 23, 24) ceramides decreased	Human plasma	(122)
MS	MS (n = 48), controls (n = 30)	Spring: C16:0, C24:0 ceramides and ceramide (d18:1/18:1 OH) increased; Summer: ceramide (d18:1/18:1 OH), ceramide (d18:1/22:0 OH) and C24:0 ceramide increased; Fall: C16:0, C18:1 ceramides and ceramide (d18:1/18:1 OH) increased; Winter: C12:0, C16:0, C18:1, C24:0 ceramides and ceramide (d18:1/22:0 OH) increased	Human serum	(128)

EDSS, expanded disability status scale; OND, other neurological disease.

Myriocin, a natural product isolated from the culture medium of *Isaria sinclairii*, is a potent immunosuppressive agent that inhibits serine palmitoyltransferase (SPT) (130, 131, 132), the first enzyme in the de novo ceramide synthesis pathway (Fig. 1). Chemical modification of myriocin led to the development of a more effective compound, 2-amino-2-[2-(4-octylphenyl) ethyl] propane-1,3-diol hydrochloride (FTY720), which has enhanced immunosuppressive activity. Unlike myriocin, FTY720 does not act directly on SPT. Instead, it serves as a prodrug that is rapidly phosphorylated by sphingosine kinase 2 to form FTY720 phosphate. This phosphorylated form binds with high affinity to four S1P receptors (S1P1, S1P3, S1P4, and S1P5), functioning as a receptor agonist (133, 134). Specifically, FTY720 prevents lymphocyte egress from the lymph nodes, thereby lowering the number of circulating lymphocytes in the peripheral blood (135). This mechanism underpins the clinical efficacy of FTY720, also known as fingolimod, which significantly reduces relapses and slows disability progression in patients with MS (136). Approved by the US Food and Drug Administration in 2010 for the treatment of MS, FTY720 highlights the potential of targeting ceramide-related pathways in managing autoimmune diseases.

### Cancer

Ceramide metabolism plays a critical role in influencing the proliferation, growth, and survival of cancer cells. In addition, ceramide is critical for exosome formation and function, and as a component of lipid rafts, it regulates signaling pathways that reprogram cancer cells and their microenvironment (137, 138). Variations in ceramide fatty acid chain length contribute to their distinct functions in cancer pathogenesis. For example, C16 ceramide has been implicated in cancer cell proliferation, whereas C18:0 ceramide is involved in cell death and growth (139, 140). The levels of C16:0, C24:0, and C24:1 ceramides in the tumor tissues of head and neck squamous cell carcinoma (HNSCC) patients are significantly higher than those in normal tissues (141). In contrast, the levels of C18:0 ceramides are significantly lower and are associated with an increased risk of lymphovascular invasion and pathological lymph node metastasis (141). C18:0 and C16:0 ceramides, which are primarily synthesized via CerS1 and CerS6, respectively, exert opposite proapoptotic and prosurvival effects in human HNSCC. The overexpression of CerS6 and the subsequent accumulation of C16:0 ceramides were observed to reduce endoplasmic reticulum stress and inhibit apoptosis in HNSCC cells (142). These data suggest that the homeostasis between C18:0 ceramide and C16:0 ceramide can regulate HNSCC progression.

Total ceramide levels in breast cancer (BC) tissue are significantly higher than those in normal breast tissue (143, 144, 145). Kanto *et al.* (146) reported that tumor cell supernatants enriched with C16:0 and C24:0 ceramides induced apoptosis in dendritic cells, thus enabling tumor cells to evade the immune surveillance system. Schiffmann *et al.* (145) showed significant increases in C16:0, C24:0, and C24:1 ceramides in malignant breast tumor tissues compared with benign and normal tissues, suggesting an association between increased ceramide levels of varying chain lengths and BC development. Within BC tissues, enhanced levels of substrates and enzymes involved in the ceramide synthesis pathways, such as glucosylceramidase, sphingomyelinase, and dihydroceramide desaturase 1, have been observed. Gene expression analysis further suggested that upregulated ceramide-generating enzymes were correlated with poorer survival outcomes in BC patients (144). Changes in ceramide levels are also evident in the serum of ovarian cancer patients (147, 148), with increases in C16:0, C18:0, C20:0, and C24:1 ceramides, alongside decreases in C23:0 and C24:0 ceramides (149) (Table 4). In HeLa cell lines, overexpression of CerS5 increased the level of C16:0 ceramide, whereas overexpression of CerS2 increased the levels of C22:0 and C24:0 ceramides (156). In summary, disturbances in ceramide metabolism are a hallmark of cancer, with ceramide levels generally upregulated in circulation and/or tumor tissues. However, it is noteworthy that C18:0 ceramide, known for its proapoptotic effects, has been shown to be downregulated in tumor tissues from patients with HNSCC (140). The levels of C18:0 ceramide in colorectal cancer and hepatocellular carcinoma disease are inconsistent, and there are upregulated (151, 153, 154) and downregulated (150, 152, 155) conditions (Table 4).

**Table 4:** Summary of studies assessing ceramide levels in patients with cancers

Cancer	Elevated ceramide	Reduced ceramide	Type of sample	References
HNSCC	C16:0	C18:0	Human HNSCC tumor tissue	(140)
HNSCC	C16:0, C24:0, C24:1	C18:0	Human HNSCC tumor tissue	(141)
BC	C16:0, C18:0, C20:0, C24:0, C24:1, C16:0/C18:0, C24:0/C18:0 *CERS2*, *CERS4* and *CERS6* mRNA were elevated		Human breast tissue	(145)
BC	C16:0, C18:1, C20:0, C22:0, C24:1, C24:0, C26:1		Human breast tissue	(143)
BC	C14:0, C16:0, C18:1, C18:0, C20:0, C22:0, C24:1, C24:0, C26:1, C26:0		Human breast tissue	(144)
OC	C16:0, C18:1, C18:0 (plasma); C16:0, C18:1, C18:0, C24:1, C24:0 (ovarian tissue)		Human plasma samples and ovarian tissue	(147)
OC	C16:0, C18:0, C20:0, C24:1	C23:0, C24:0	Human serum samples	(149)
OC	C16:0, C22:0, C24:0		Human serum samples	(148)
CRC	C16:0, C24:0, C24:1, *CERS1*, *CERS2*, *CERS5* and *CERS6* mRNA were elevated.	C18:0, C20:0	Human colorectal cancer tissue	(150)
CRC	C16:0, C18:0, C18:1, C24:1		Human serum samples	(151)
CRC	C14:0	C18:0, C20:0	Human colorectal cancer tissue	(152)
HCC	C16:0, C18:0, C20:0, C24:0		Human serum samples	(153)
HCC	C14:0, C16:0, C18:1, C18:0, C20:0, C22:0, C24:1, C24:0, C26:1, C26:0, *DES1*, *GBA*, *GBA2*, *SMPD2* and *SMPD4* mRNA were elevated		Human hepatocellular carcinoma tumor tissue	(154)
HCC	C12:0, C16:0, C18:1, C24:1, *DES1*, *DES2*, and *CERS6* mRNA were elevated	C18:0, C20:0, C24:0, *CERS1* and *CERS4* mRNA were reduced	Human hepatocellular carcinoma tumor tissue	(155)

CRC, colorectal cancer; DES, dihydroceramide desaturase; GBA, glucocerebrosidase; HCC, hepatocellular carcinoma; OC, ovarian cancer; SMPD, sphingomyelin phosphodiesterase.

## Dietary interventions

**Fig. 3:**
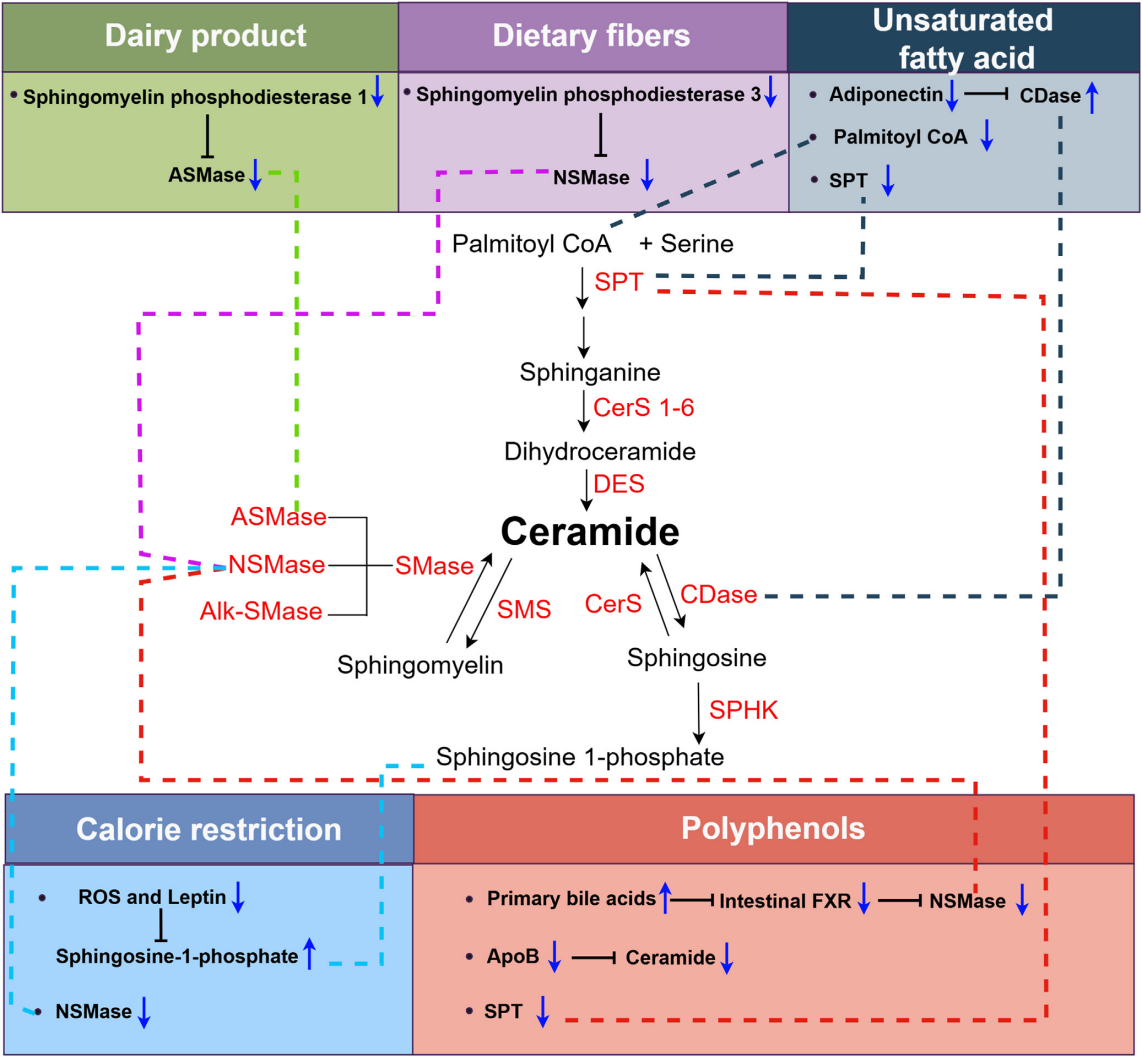
Mechanisms by which nutritional interventions alter ceramide metabolism (created by Figdraw 2.0). Key dietary nutrients/components, such as the dairy products, dietary fibers, unsaturated fatty acids, polyphenols, and calorie-restricted diet, are depicted in relation to their influences on ceramide synthesis and degradation pathways. The blue solid arrows represent the upregulation or downregulation of biochemicals or enzymes. Alk-SMase, alkaline sphingomyelinase; ApoB, apolipoprotein B; CDase, ceramidase; DES, dihydroceramide desaturase; FXR, farnesoid X receptor; ROS, reactive oxygen species; SMase, sphingomyelinase; SMS, sphingomyelin synthase; SPHK, sphingosine kinase.

Potential drug targets in ceramide metabolism for treating CVDs (157), cancers (158), and neurological disorders (159) have been previously reviewed. In addition, Tsukamoto *et al.* (160) provided a comprehensive review of small-molecule inhibitors targeting ceramide biosynthesis with the potential for therapeutic applications. However, challenges remain in developing drugs targeting ceramide metabolism. The complexity of ceramide biosynthesis and the interactions among various ceramide species and regulatory pathways in different cell types complicate treatment outcomes (29). Furthermore, research into the pharmacokinetics, pharmacodynamics, and off-target effects of ceramide-targeted therapies is essential to ensure safety and tolerability across diverse patient populations (161, 162). Dietary intervention offers an alternative approach for managing circulating ceramide levels. A healthy dietary pattern may provide a biologically meaningful approach for modulating ceramide levels in affected individuals, particularly when combined with available therapeutic treatment in the future. A schematic overview detailing the mechanisms underlying nutritional interventions aimed at modulating circulating ceramides is depicted in Fig. 3.

### MD and other healthy dietary patterns

The MD, characterized by minimally processed and plant-derived foods, is associated with a wide range of health benefits (163, 164). Studies have investigated the effects of various dietary patterns, including the MD, on the ceramide response in humans. In the PREvencion con DIeta MEDiterranea trial, adults at high CVD risk were randomly assigned to receive an MD supplemented with extra virgin olive oil (MD + EVOO), an MD supplemented with nuts (MD + nuts), or a control diet low in total fat, with an average follow-up duration of 4.8 years. The results indicated that a traditional MD enriched with EVOO or nuts has the potential to alleviate the harmful effects of elevated plasma ceramide concentrations on CVD risk (165). In the Framingham progeny cohort study, individuals in the highest quartile of Dietary Guidelines Adherence Index compliance (evaluates how well individuals adhere to the Dietary Guidelines for Americans) exhibited lower concentrations of C16:0, C22:0, C24:0 ceramides, and C22:0/C16:0 ceramide ratios. Similarly, higher quartiles of the Mediterranean-style diet score correlated with significantly reduced concentrations of C16:0 and C22:0 ceramides (166). In the ADIRA crossover trial, 50 rheumatoid arthritis patients followed a 10-week Mediterranean-style diet intervention and a Western-style control diet, separated by a 4-month washout period. After the MD intervention, the serum levels of the ceramide species, C16:0, C18:0, and C24:0, were significantly lower than those following the control diet (167). The health effects of the Nordic Diet, a regional alternative to the MD, have also attracted great interest (168, 169). Maria *et al.* (170) observed statistically significant alterations in plasma lipids, including decreases in ceramides, in the healthy Nordic diet group compared with the control group (average Nordic diet with a limited amount of fruits, vegetables, and berries). Similarly, findings from the FRUVEDomic pilot study showed that an 8-week nutritional intervention with fruit- and vegetable-rich diets could lower circulating ceramide levels (171). Djekic *et al.* (36) also demonstrated that a 4-week vegetarian diet lowered ceramide C16:0 levels and cardiometabolic risk factors compared with an isocaloric meat-containing diet.

The foundation of these healthy diets is plant-derived foods, which indicates that their beneficial effects may be derived from key components such as unsaturated fatty acids (172, 173), dietary fibers (174), and polyphenols (175). The effects of these active nutrients on ceramides are discussed in the following sections.

### Calorie-restricted diet

Calorie restriction (CR) is defined as a sustained reduction in daily energy intake from the preintervention energy requirement while maintaining adequate nutrient provision (176, 177). This dietary regimen has been shown to enhance insulin sensitivity and lower the incidence of diabetes and associated metabolic disorders (37). A 16-week CR intervention has been reported to reduce total skeletal ceramide levels in obese individuals (178). Similarly, a 6-week CR diet intervention study revealed significant improvements in gut microbial gene richness, accompanied by a notable 39% and 35% decreases in serum C18:2 and C18:1 ceramides, respectively (179). CR diets reduce dietary fat intake, and long-term decreases in sphingolipid precursor (saturated fatty acids) may be responsible for reduced de novo ceramide synthesis (180). An animal study demonstrates that redox-sensitive NSMase plays an important role in the dysregulation of SM turnover in the hippocampus and neocortex in aged rats, and the CR diet mainly prevents the age-dependent accumulation of ceramides by targeting NSMase (180). Reactive oxygen species can act as a key regulatory factor in the interconversion of ceramide and S1P. Under conditions of excessive oxidative stress, sphingosine kinase 1 is downregulated, leading to decreased S1P levels and the subsequent accumulation of ceramides (181). Importantly, CR has been shown to mitigate reactive oxygen species formation, thereby potentially reducing ceramide production (182).

### Dairy product-based interventions

Dairy products, such as milk, cheese, and yogurt, are considered sources of essential vitamins, minerals, and/or high-quality protein (183). Epidemiological evidence suggests a favorable correlation between dairy consumption and improved cardiometabolic risk factors (184). Qiu *et al.* (185) reported that the efficacy of replacing soda with low-fat milk in improving atherogenic dyslipidemia and other cardiometabolic risk factors in overweight or obese adolescent males was investigated. Lipid analysis revealed that milk consumption significantly reduced plasma glucosylceramide (d18:1/C16:0) and lactosylceramide (d18:1/C16:0 and d18:1/C18:0) levels. In a 4-week randomized controlled trial, 58 postmenopausal women consumed cream cheese enriched with milk polar lipids daily. The results showed that milk polar lipids reduced the serum atherogenic ceramide C24:1, which is potentially linked to decreased RNA expression of sphingomyelin phosphodiesterase 1 (186). Despite the high intake of sphingolipids provided by milk polar lipids, the concentrations of sphingomyelins and ceramides in gut-derived chylomicrons decreased, suggesting a plausible mechanism underlying the beneficial effects of dairy products.

### Unsaturated fatty acids

Unsaturated fatty acids offer well-documented benefits against a variety of diseases, including coronary artery disease (187), diabetes (188), cancer (189), and autoimmune diseases (190). Alterations in the dietary composition of fatty acids have been shown to modulate systemic and cellular ceramide metabolism in humans (191). For example, excessive intake of saturated fats led to increased levels of ceramides in both the circulation and liver (192). Palm oil-derived saturated fatty acids significantly induce elevated liver fat and serum ceramides, whereas dietary PUFAs prevent hepatic fat accumulation and reduce ceramide levels and hyperlipidemia during excess energy intake in overweight individuals (193). In female subjects, a diet rich in palmitic acid resulted in increased ceramide levels and muscle mass, whereas an oleic acid-rich diet corresponded to reduced serum and muscle ceramide concentrations, along with decreased molecular biomarkers of inflammation and oxidative stress (191). In a 2-week, double-blind, crossover, randomized controlled trial, healthy normal-weight participants were administered chocolate sauce snacks containing different fat types, i.e., EVOO rich in monounsaturated fatty acids or palmitic acid. The results showed that consumption of EVOO-rich chocolate sauce yielded lower plasma C16 ceramide levels and ratios of C16/C22:0 and C16/C24:0 compared to palmitic acid-supplemented chocolate sauce (194). Similarly, a double-blind randomized controlled trial investigating n-3 long-chain unsaturated fatty acid supplementation in pregnant women showed alterations in the plasma metabolome of offspring, manifesting as reduced ceramide and sphingolipid levels containing 18:0 and 22:0 fatty acyl chains (195). Further, Lankinen *et al.* (39) studied the effects of fatty fish, lean fish, or lean meat consumption on plasma lipid profiles in patients with coronary heart disease and observed a significant reduction in ceramides in the group that consumed fatty fish compared with those consuming lean fish or control diets. Walnuts are particularly rich in PUFAs such as alpha-linolenic acid (196). Tuccinardi *et al.* (197) found that walnut consumption led to significant reductions of ceramides, hexosylceramides, and SM in obese individuals. These effects could result from the elevation of adiponectin (198), which can promote ceramide reduction through increased ceramidase activity (199) (Fig. 3). SPT catalyzes the de novo synthesis of ceramides, and its activity is heavily dependent on the availability of palmitoyl-CoA. Because SPT preferentially utilizes fatty acyl-CoAs with 16 ± 1 carbon atoms, other fatty acids may inhibit its activity by competing for the CoA pool (200). Unsaturated free fatty acids exhibit preventive effects against excess ceramide accumulation stimulated by saturated free fatty acids (172), in which SPT may play a key role (173). It should be noted that a diet rich in unsaturated fatty acids has been shown to elevate the plasma levels of C20:0 and C24:1 glucosylceramides while reducing ceramides such as C16:0, C22:0, C24:0, and C26:0. This reduction in ceramides has been hypothesized to result from increased glycosphingolipid production. Taken together, these findings suggest that unsaturated fatty acid supplementation or the consumption of food rich in unsaturated fatty acids can reduce circulating ceramide levels.

### Dietary fibers

Dietary fiber, a category of nondigestible carbohydrates primarily composed of polysaccharides derived from plant foods, is broadly classified into water soluble and insoluble fibers (201). Long-term consumption of dietary fibers is generally associated with metabolic health benefits (202). In a clinical intervention trial, a comprehensive multiomics analysis was conducted to assess the effects of arabinoxylan-oligosaccharide-enriched diet on overweight individuals with metabolic syndromes. The results showed that arabinoxylan-oligosaccharide intake significantly reduces plasma ceramide concentrations (203). The gut microbiota may represent further participants in the link between diet, lipid metabolism, and metabolic health. Vijay *et al.* (38) evaluated the impact of inulin dietary fiber (water soluble) on gut microbiome composition and CVD risks in healthy participants. This study indicated that reductions in specific ceramide ratios, including the C18:1/C16:0, C18:0/C24:0, and C18:1/C24:1, were associated with increased abundance of *Bifidobacterium* and *Coprococcus 3*, as well as short-chain fatty acids after inulin intake, highlighting the potential of prebiotic dietary fiber in modulating ceramide levels, partly attributed to alterations in the gut microbiome composition and metabolite production. Mechanistically, dietary fibers such as inulin can modulate the gene expression of *Smpd3* and regulate the activity of NSMase2, a crucial enzyme involved in the hydrolysis of SM to ceramide (174, 204) (Fig. 3).

### Polyphenols

Polyphenols constitute a diverse group of phenolic compounds that are abundant in plant foods and are known for their manifold health-promoting effects (205). Epidemiological evidences indicate that higher dietary intake of polyphenols may decrease the incidence rate and mortality of CVD (206), T2D (207) and hypertension (208). In a randomized, double-blind, placebo-controlled trial, 176 subjects with dyslipidemia were allocated to receive a placebo or anthocyanin at doses of 40, 80, or 320 mg/day for 12 weeks. Compared with placebo, the intake of 320 mg/d anthocyanins effectively reduced plasma C16:0 and C24:0 ceramide levels (40). Furthermore, a blueberry anthocyanin-rich extract effectively decreased serum ceramide levels in high-fat diet-fed mice by downregulating the genes of enzymes involved in de novo ceramide synthesis and SM hydrolysis, i.e., SPT, CerS, sphingolipid 4-desaturase, and sphingomyelin phosphodiesterase (209). Tveter *et al.* reported that supplementation with proanthocyanidin-rich grape polyphenol extract decreased expression of ceramide biosynthesis genes *Smpd3*, serine palmitoyltransferase long chain base subunit 2 (*Sptlc2*), and *CerS4* in the liver and gut tissues of mice. These effects may be associated with the regulation of bile acids by polyphenols and the inhibition of intestinal farnesoid X receptor (210). Notably, ceramides are predominantly transported by apolipoprotein B-rich lipoproteins (211, 212). Dietary anthocyanins could potentially stimulate the clearance and catabolism of apolipoprotein B-containing lipoproteins, thereby hindering the secretion and transport of ceramides (40).

White wine contains numerous phenolic compounds, including tyrosol, an antioxidant with potential cardioprotective properties (213). In a study examining the impact of white wine enriched with tyrosol on circulating ceramide levels in a population at high risk of CVD, it was found that regular consumption of white wine consumption led to a significant reduction in the ceramide ratio of C16:0/C24:0. Notably, intervention with the white wine supplemented with tyrosol resulted in decreased ceramide ratios of C16:0/C24:0, C18:0/C24:0, and C24:1/C24:0. These changes are associated with improved endothelial function as indicated by the reactive hyperemia index and the augmentation index (214).

Diet influences the sphingosine and fatty acid composition of ceramide by modulating the availability of substrates and affecting metabolic pathways. Healthy dietary patterns are generally associated with lower concentrations of very long-chain ceramides. For example, a cross-sectional study of middle-aged adults found C22:0 ceramide was the only one ceramide significantly associated with the 2015 Healthy Eating Index, and it was also inversely associated with the saturated fat and added sugar intakes (215). A randomized clinical trial found that a healthy Nordic diet led to statistically significant reductions in the concentrations of very long-chain ceramides, including C22:0, C23:0, and C24:0, after 12 weeks (170). Furthermore, studies have reported that a higher cumulative Mediterranean-style diet score was inversely associated with concentrations of the C16:0 and C22:0 ceramides (166, 216). This effect is thought to result from reduced de novo lipogenesis due to lower saturated fat intake. However, it should be noted that in the PREDIMED trial, no differences were observed in the concentrations of ceramides C16:0, C22:0, C24:0, and C24:1 in the MD intervention groups compared with the control diet group (165), likely because the control group also followed a variant of the MD (217). Specific dietary components, such as walnuts, have been linked to decreased total ceramides and increased sphingosine concentrations, which could be related to the enhanced catabolism of ceramide (197). These findings suggest that diet and dietary components can distinctly affect ceramide and sphingosine profiles. A combination of dietary interventions may be more effective in achieving biologically meaningful reductions in circulating ceramide. Therefore, further studies are needed to elucidate the effects of various dietary pattern combinations on specific ceramide species and their broader implications for health.

## Conclusions and future perspectives

Ceramides play a significant role in the pathogenesis of various conditions, including cardiometabolic diseases, neurological disorders, autoimmune diseases, and cancers. Understanding ceramide function is crucial for developing precision interventions aimed at preventing and managing disorders associated with elevated ceramide levels. This article summarizes promising findings that suggest dietary interventions can reduce circulating ceramide levels, offering a potential anticeramide regimen. To better understand how dietary changes influence ceramide composition and contribute to disease, several key areas require further investigation: *1*) since ceramides are part of a broader sphingolipid metabolism, it is important to understand how dietary components affect related sphingolipids, such as glycosphingolipid and sphingosine-1-phosphate metabolism. Exploring these interactions will help clarify the effects of diet on overall sphingolipid homeostasis and its implications in inflammation and cell signaling pathways; *2*) studies are needed to confirm how dietary patterns consistently influence ceramide, including their impact on specific ceramide species in distinct organs and cellular compartments; and *3*) although dietary components and patterns are known to regulate ceramide levels, further research is needed to uncover the underlying mechanisms, including the potential synergistic effects of these components and patterns, at the enzymatic and transcriptomic levels. Such insights could enable the development of tailored dietary plans to enhance intervention outcomes and improve disease management.

## Conflict of interest

The authors declare that they have no conflicts of interest with the contents of this article.
